# Land Use and Land Cover Change in the Qinghai Lake Region of the Tibetan Plateau and Its Impact on Ecosystem Services

**DOI:** 10.3390/ijerph14070818

**Published:** 2017-07-21

**Authors:** Jian Gong, Jingye Li, Jianxin Yang, Shicheng Li, Wenwu Tang

**Affiliations:** 1 Department of Land Resource Management, School of Public Administration, China University of Geosciences (Wuhan), 388 Lumo Road, Hongshan District, Wuhan 430074, Hubei, China; jingye.li@outlook.com (J.L.); yangjianxinjian@163.com (J.Y.); lisc@cug.edu.cn (S.L.); 2 Key Labs of Law Evaluation of Ministry of Land and Resources of China, 388 Lumo Road, Hongshan District, Wuhan 430074, Hubei, China; 3 Department of Geography and Earth Sciences, The University of North Carolina at Charlotte, 9201 University City Blvd., Charlotte, NC 28223, USA; wenwutang@gmail.com; 4 Center for Applied Geographic Information Science, The University of North Carolina at Charlotte, 9201 University City Blvd., Charlotte, NC 28223, USA

**Keywords:** land use and land cover change, ecosystem service, Markov-Cellular Automata, value assessment, sensitive analysis, Tibetan Plateau

## Abstract

Exploration of land use and land cover change (LULCC) and its impacts on ecosystem services in Tibetan plateau is valuable for landscape and environmental conservation. In this study, we conduct spatial analysis on empirical land use and land cover data in the Qinghai Lake region for 1990, 2000, and 2010 and simulate land cover patterns for 2020. We then evaluate the impacts of LULCC on ecosystem service value (ESV), and analyze the sensitivity of ESV to LULCC to identify the ecologically sensitive area. Our results indicate that, from 1990 to 2010, the area of forest and grassland increased while the area of unused land decreased. Simulation results suggest that the area of grassland and forest will continue to increase and the area of cropland and unused land will decrease for 2010–2020. The ESV in the study area increased from 694.50 billion Yuan in 1990 to 714.28 billion Yuan in 2000, and to 696.72 billion Yuan in 2020. Hydrology regulation and waste treatment are the top two ecosystem services in this region. The towns surrounding the Qinghai Lake have high ESVs, especially in the north of the Qinghai Lake. The towns with high ESV sensitivity to LULCC are located in the northwest, while the towns in the north of the Qinghai Lake experienced substantial increase in sensitivity index from 2000–2010 to 2010–2020, especially for three regulation services and aesthetic landscape provision services.

## 1. Introduction

In this article, we focus on studying the impact of land use and land cover change on ecosystem services in Qinghai Lake region of the Tibetan Plateau. The Tibetan Plateau is the source of Asia’s major rivers, including Yangtze River, Yellow River, and Lancang/Mekong River. The Tibetan Plateau (often referred to as the third Pole besides the North and South Poles) plays a significant role in regulating the earth’s climate change [1]. The ecosystem of the Tibetan Plateau is fragile and very sensitive to global change and human intervention. Due to increased anthropogenic activities and global climate change, environmental conditions of the Tibetan Plateau have changed significantly in the past few decades [2,3,4], including grassland degradation, permafrost degradation, and biodiversity loss [5,6]. The Qinghai Lake region, as a transitional zone between the arid northwest and the Tibetan Plateau in China, is an ecological security barrier that prevents eastern China from desertification [7]. The Qinghai Lake region has developed rapidly over the past 20 years under the influence of Western Development Policy implemented by the Chinese government. The Qinghai Lake region has been experiencing intensified human land use activities, which has a substantial influence on the ecosystems of the Tibetan Plateau. However, only a few studies were conducted in terms of land use and land cover change and its impacts on the ecosystem of this internationally recognized region [8]. Often time, studies reported in the literature were only conducted at plateau or provincial scale [9,10].

Anthropogenic land use activities produce significant impact on natural environment and have modified earth surface dramatically [11,12,13,14,15]. Ecosystem is not only the living environment of human beings, but also provides a series of services for us [16,17,18]. Increased land use intensity and frequent conversion among different land use types have modified the structure of ecosystems, leading to change in the functions of these ecosystems and values of associated ecosystem services [19,20]. In addition, with the emergence of growing global environmental problems and demand for green and sustainable development, land use and cover change (LULCC) and its impact on ecosystem service value (ESV) have captured increasing attention [16,21,22,23,24]. International or regional organizations or initiatives, including the Global Land Programme [25], Millennium Ecosystem Assessment [26], MAES (Mapping and Assessment of Ecosystems and their Services) in Europe [18], TEEB (The Economics of Ecosystems and Biodiversity) and IPBES (Intergovernmental Science-Policy Platform on Biodiversity and Ecosystem Services), the Ecological Society of America [27], the Ecological Society of British [28], and the European Union’s Biodiversity Conservation Strategy [29,30] further pushed the study of ecosystem service assessment.

In China, scholars have conducted substantial work on ecosystem service study [31,32,33,34]. Based on questionnaire survey of more than 200 scholars with ecological backgrounds, Xie et al. [35] proposed a representative ESV evaluation unit system and updated its parameters later [36,37]. Recently, the report on China’s first national ecosystem assessment (2000–2010) indicated that national conservation policies in China contributed significantly to increases in ecosystem services [33]. However, in terms of the studies at local to regional scales, more work is still needed [38]. Because of lack of data that capture fine-level details, studies at, for example, township level are scarce, not to mention the projection of future trends at this level [39,40].

According to the proposed government policy, Four zones, Two belts, and One line Development Plan in Planning of Main Functional Area in The Qinghai Province (2008–2020), the Qinghai Lake region has been identified as a modern high-efficiency animal husbandry production base and a demonstration area where human and nature live in harmony. For the purpose of sustainable development, it is urgent to study the relationship between land use activities and its ecological effects in this region.

Thus, in this study, we investigate the spatially explicit characteristics of LULCC over three time periods (1990, 2000, and 2010) in the Qinghai Lake region and future LULCC patterns simulated from Markov-Cellular Automata (Markov-CA) model. Based on the previous work by Xie et al. [37], we evaluate quantitatively the impact of LULCC on ESV at township scale for 1990–2020. Last, to identify the ecologically sensitive area to anthropogenic activity, we carry out the sensitivity analysis of ESV to LULCC.

## 2. Materials and Methods

### 2.1. Study Area

**Figure 1:**
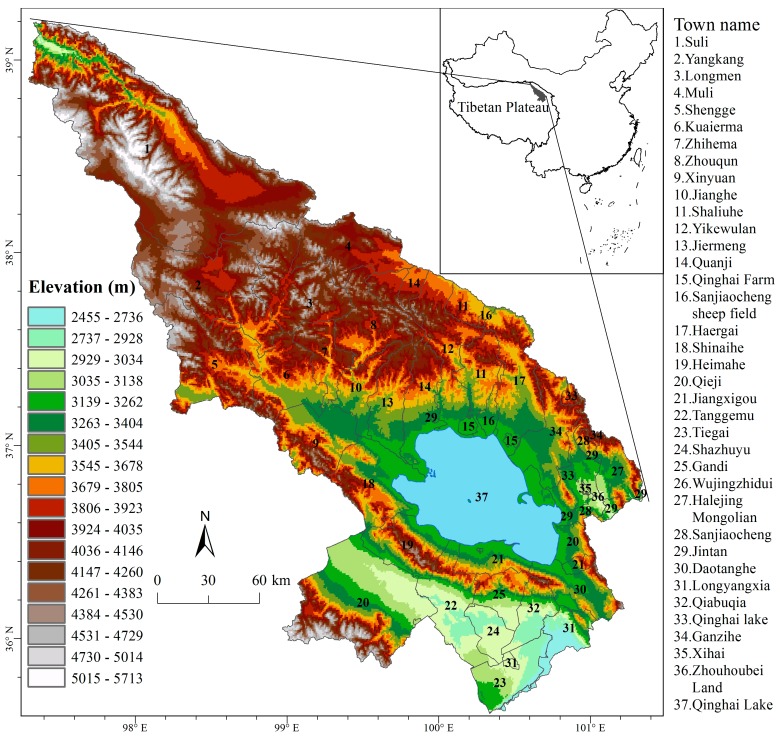
Map of the study area: the Qinghai Lake region, China (The elevation data is provided by International Scientific & Technical Data Mirror Site, Computer Network Information Center, Chinese Academy of Sciences (http://www.gscloud.cn). The vector boundary data for county and township are provided by National Administration of Surveying, Mapping and Geoinformation of China (http://en.nasg.gov.cn/)).

Our study area is the Qinghai Lake region, which is located in the northeastern part of Qinghai Province and lies between latitudes 36° and 38° N and longitudes 99° and 103° E (Figure 1). Our study region includes 4 counties within two prefectures: Haiyan County and Gangcha County in Haibei Prefecture, Tianjun County in Haixi Prefecture, and Gonghe County in Hainan Prefecture. In total, there are 38 towns in this region. The total area of our study region is 55,700 km^2^. Qinghai Lake is the largest salt lake in China, which is an important international wetland. The elevation of the study region ranges from 2403 m to 5814 m. Grassland is the major land cover type, accounting for about 63% of the study area [8].

### 2.2. Data

We obtained land use and land cover data at a spatial resolution of 30 m by 30 m for 1990, 2000, and 2010 from Landsat TM 5 and 7 satellite images (from US Geological Survey; http://earthexplorer.usgs.gov). Track numbers are 132, 133, 134, and 135, and line numbers are 33, 34, and 35. System radiation correction and geometric correction were applied to these Landsat images. Combining field investigation and visual inspection, we conducted classification on Landsat imagery for land use and land cover information in our study region. As a result, we have seven primary land cover types, including cropland, woodland, grassland, wetland, waterbody, construction land, and unused land. The overall classification accuracy was 83.5% and the Kappa coefficient 0.81. Besides, the socioeconomic data, including crop yield, wheat sowing area, and wheat yield price were obtained from tables named major economic indicators of counties in Qinghai statistics yearbook 1990 [41], 2000 [42], and 2010 [43].

### 2.3. Methods

**Figure 2:**
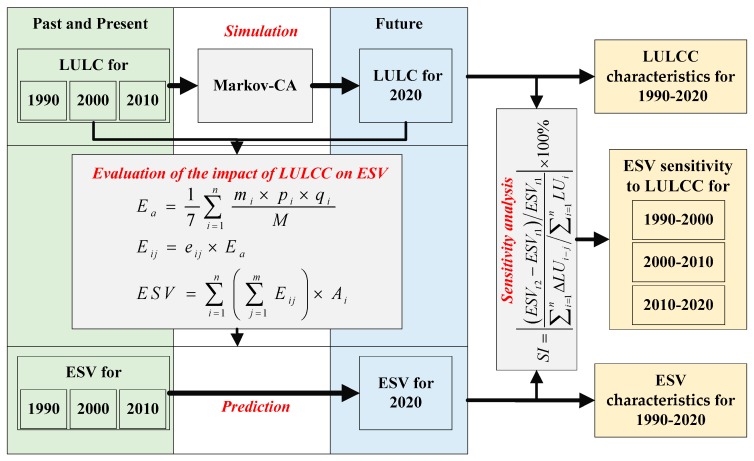
Framework of this study. LULC: land use and land cover; LULCC: land use and land cover change; ESV: ecosystem service value.

There are three steps in this study. First, based on the satellite-derived land cover data of the Qinghai lake region for 1990, 2000, and 2010 and utilizing the Markov-CA model, we simulated the LULCC of the region for 2020. Then, employing the equivalent factors of Xie et al. [37] which is localized based on the parameters of Costanza et al. [44], we evaluated the impacts of LULCC on ESV and obtained the spatial patterns of ESV at township scale for the four years. In total, 9 types of ES functions were analyzed at township scale. The township scale is valuable for local governments to make conservation planning. Because ecosystem services are not adequately quantified in comparison with economic services and manufactured capital, they are often given too little weight in policy decisions. This may ultimately compromise the human-related sustainability in the biosphere [44]. Thus, we attached a price tag on ecosystem services in this study. Finally, we analyzed the sensitivity of ESV to LULCC using the methods of [10,19], which is updated in this study (Figure 2).

#### 2.3.1. Land Use and Land Cover Simulation

We used a Markov-CA model to simulate the land cover change in our study region. Markov chain is a form of stochastic process that makes use of probabilities of the previous state of system and the transition probability between alternative states to forecast future trend. However, the Markov model is limited in directly simulating the spatial pattern of land use, which can be enhanced using Cellular Automata (CA). The CA model is a simulation approach that relies on discrete space, time, and state to represent spatial dynamics of geographic phenomena [45]. A typical CA model consists of cells, states, rules, and neighborhoods, which can be expressed as follows:

CA = (*L^d^*, *S*, Δ, *F*) (1)

where *L* is the space where cells are located. *d* is the dimension of the cellular space. *S* is a finite set of discrete states. Δ is the neighborhood set of a cell. *F* denotes the rule of state transition of cells, which determines the state of a cell next step according to the current state of the cell itself and its neighboring cells. When utilizing the Markov-CA model to simulate the land use change, *L^d^* is usually a two-dimensional grid of cells driven by rules. Each grid cell represents a land unit. *S* is the set of land use types of the study area. The rule *F* is denoted by land use transition area matrix and the conditional probability calculated by the Markov model. To determine the state transition of cells and the dynamic evolution of system state, the CA model was used based on the initial state of the system via iterations.

In this study, based on the 1990 and 2000 land use data and the Markov model, we can estimate the land use area for 2010 and simulate the spatial patterns based on conditional probability for each land use type [46]. Utilizing the land cover map for 2000 as initial conditions and the probability surfaces as land suitability map, we simulated the land cover patterns of the study area for 2010 using CA iterations (5 × 5 von Neuman neighborhood rule). The spatial resolution of the simulation model is set as 30 m by 30 m and the landscape size in terms of number of rows and columns is: 13,082 by 12,487. The temporal resolution of the model is 1 year, corresponding to one iteration in the simulation model. We compared the simulated land cover map with the empirically observed data. The Kappa coefficient between simulated and empirical land cover patterns is 0.66, suggesting that the simulation accuracy is relatively high. Therefore, our model can provide effective support for the simulation of LULCC in the study area. Then, the land use area for 2020 was projected by Markov model using land use data for 2000 and 2010 [46]. Based on land cover map for 2010, we obtained the transition probability for each land use type in 2020. Using the 2010 land cover map as initial conditions and land suitability map generated from Markov transition probability matrix, the land cover for 2020 was simulated using the CA model.

#### 2.3.2. Analysis of Land Use and Land Cover Change

We used land use transition matrix and land use dynamic degree to analyze the characteristics of land use change. Land use transition matrix reflects the structural characteristics of land use change, i.e., the direction and quantity characteristics of LULCC. Land use dynamic degree includes Single Land Use Dynamic Degree (SLUDD) and Comprehensive Land Use Dynamic Degree (CLUDD). SLUDD (denoted as *K* in Equation (2)) reflects the change rate of a single land use type while CLUDD (denoted as *R* in Equation (3)) evaluates the overall situation of land use change rate [47]:

(2) $$K=\frac{U_{b}-U_{a}}{U_{a}}\times \frac{1}{T}\times 100\%$$

(3) $$R=\left[\frac{\sum_{i=1}^{n}\Delta LU_{i-j}}{\sum_{i=1}^{n}LU_{i}}\right]\times \frac{1}{T}\times 100\%$$

where *U_a_* and *U_b_* represent the land use area of a single land use type at beginning and end of the study period. *T* is the range of the study period. If *K* is greater than 0, the study region is in the land expansion period (otherwise, land shrinkage). *LU_i_* represents the initial area of land use type *i*. *ΔLU_i-j_* is the area of land use type *i* converted to land use type *j* during the study period.

#### 2.3.3. Evaluation of the Impact of Land Use and Land Cover Change on Ecosystem Service Value

Based on the study by Costanza in 1997 [44], Xie et al. [37] carried out a questionnaire survey among 299 Chinese ecologists, and developed a new evaluation system for ESV. This system includes the equivalents of the values of nine ecological services in six ecosystems. The economic value of national average grain yield of cropland with an area of 1 hm^2^ is defined as one unit. Other equivalent factors are the contribution values relative to food production service of cropland. These equivalent factors were updated in 2008 as more ecologists were surveyed (Table S1). In this study, we used the updated factors for the evaluation of ESV.

The economic value of the food production service per unit area of cropland was calculated via the following equation [48]:

(4) $$E_{a}=\frac{1}{7}\sum_{i=1}^{n}\frac{m_{i}\times p_{i}\times q_{i}}{M}$$

where *E_a_* denotes the price of the food production service per unit area of cropland (unit: Yuan/hm^2^/a). *i* denotes crop types (including wheat, coarse cereal, and tuber crop). *m_i_* is the sowing area of the crop *i* (unit: hm^2^). *p_i_* is the national mean price of crop *i* (unit: Yuan/t). *q_i_* is the yield per unit area of crop *i* (unit: t/hm^2^/a). *M* is the total sowing area of all crop types (unit: hm^2^).

After determining the economic value of food production service per unit area of cropland, the unit price of each ecological service function is calculated as follows [48]:

(5) $$E_{ij}=e_{ij}\times E_{a}$$

where *E_ij_* is the unit price of ecological service *i* of ecosystem *j* (unit: Yuan/hm^2^/a). *e_ij_* denotes the equivalent factor of the unit price of the ecological service *i* in ecosystem *j*. *i* denotes the type of ecological service function. *j* refers to ecosystem type. Because construction land is transformed from other land use types under the influence of human activities, the service function of this type of ecosystem is lost and its service value is thus not considered.

Therefore, the ESV is estimated according to the following formula [48]:

(6) $$ESV=\sum_{i=1}^{n}\left(\sum_{j=1}^{m}E_{ij}\right)\times A_{i}$$

where *ESV* is the total ecological service value. *A_i_* is the area of land use type *i*. *n* is the number of land types. *m* is the number of ecosystem service function types.

#### 2.3.4. Sensitivity Analysis of Ecosystem Service Value to Land Use and Land Cover Change

Based on the elasticity theory of economics, we conducted sensitivity analysis to evaluate the sensitivity of ESV to LULCC. This analysis further reveals the ecologically sensitive area to human activity in Qinghai Lake area. Based on previous studies [10,19], the sensitivity index (*SI*) is defined as in Equation (7):

(7) $$SI=\left|\frac{\left(ESV_{t2}-ESV_{t1}\right)}{R}\right|\times \frac{1}{t_{2}-t_{1}}\times 100\%$$

where *SI* is the sensitivity index of changes in ESV in response to LULCC. *ESV_t_*_1_ is the ESV in year *t*_1_. *ESV_t_*_2_ is the ESV in year *t*_2_. *R* is the CLUDD (defined in Equation (3)). A high value of sensitivity index indicates that small change in land use will result in substantial change in ESV, and the area with high sensitivity index is very sensitive to human land use activities. Otherwise, the ESV is insensitive to LULCC.

## 3. Results

### 3.1. Analysis of Land Use and Land Cover Change

We first analyze the LULCC characteristics for 1990–2010 based on satellite-derived data. Then, based on the simulated land use/cover data for 2020, we discussed the characteristics of LULCC for 2010–2020.

#### 3.1.1. Land Use and Land Cover Change for 1990–2010

**Figure 3:**
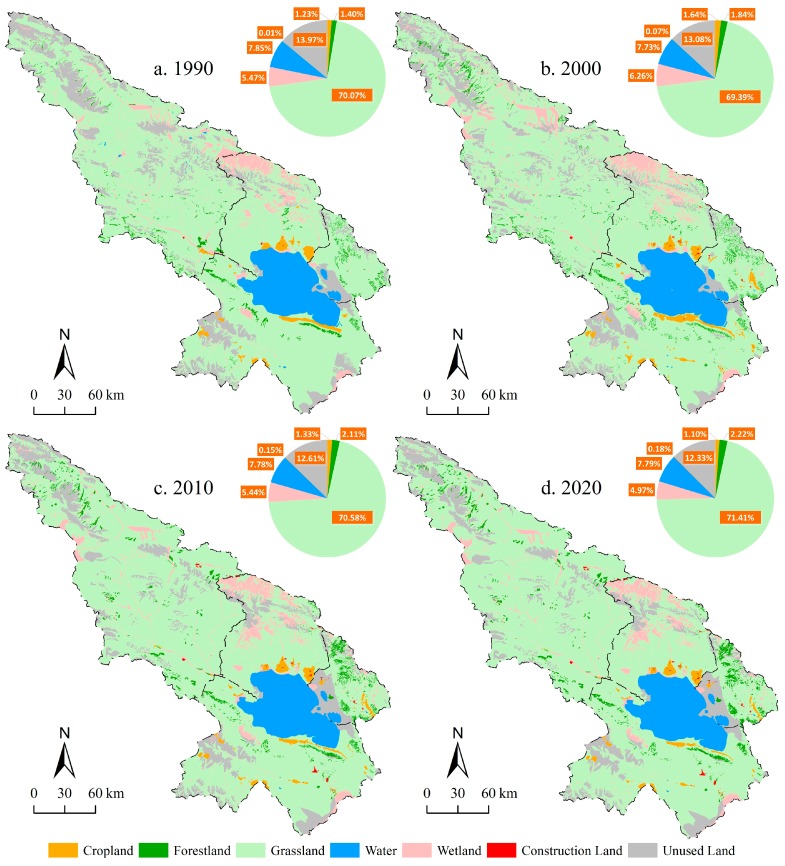
Land use/cover map for 1990, 2000, 2010, and 2020 in the Qinghai Lake region. The map for 1990, 2000 and 2010 were interpreted from Landsat TM 5 and 7 satellite images (http://earthexplorer.usgs.gov), and the map for 2020 was simulated using CA-Markov model.

The general trend of LULCC in the Qinghai Lake region for 1990–2010 was that the area of construction land, forest, grassland, and cropland increased while the area of unused land and wetland decreased (Figure 3). The SLUDD (Equation (2)) of construction land (urban areas and other settlements) for 1990–2010 was 105.05% (the largest among all land use types), followed by woodland with a SLUDD of 5.03%.

For the first decade, the CLUDD of the Qinghai Lake region was 1.61% and it could be seen that land use conversion among different types was substantial (Table 1). The area of grassland decreased by 378.23 km^2^, mainly distributed in Suli Township of Tianjun County and Ganzihe Township of Gangcha County (Figure 3a,b). The SLUDD of grassland was −0.10%, and most of the grassland were converted to unused land, wetland, and forest. Cropland increased by 227.49 km^2^ and its SLUDD was 3.32%. Most of them were converted from grassland. The area of construction land increased by 30.40 km^2^, with a SLUDD of 40.83%. Cropland and grassland were occupied by the expansion of construction land. The area of forest increased by 242.44 km^2^ (SLUDD: 3.10%.), distributed in Sanjiaocheng Township of Haiyan County, Xihai Township of Gangcha County, and Daotanghe Town of Gonghe County. The area of wetland area increased by 440.33 km^2^ (SLUDD: 1.44%), mainly distributed in Suli Township of Tianjun County, Jiermeng Township of Gangcha County, and Qieji Township of Gonghe County. The loss of unused land is 493.78 km^2^ with −0.63% of SLUDD.

The CLUDD in the Qinghai Lake region for the second decade was 1.68%. For this period, the area of grassland, forest, cropland, and construction land increased and the area of wetland and unused land decreased considerably. Due to the process of urbanization, the area of cropland decreased along with the expansion of construction land (Table S2). The implementation of ecological protection projects, including the Grain for Green Programme and the establishment of nature reserves, led to a gain of 664.66 km^2^ in terms of the area of grassland (the SLUDD was 0.17%). The grassland gain is distributed in Suli Township, Muli Town, Heimahe Township, and Jiangxigou Township (Figure 3b,c). The area of cropland and wetland decreased by 171.94 km^2^ and 454.01 km^2^, respectively, and the corresponding SLUDDs were −1.88% and −1.30%. The area of forest and construction land increased by 151.35 km^2^ and 47.81 km^2^, respectively (the corresponding SLUDDs: 1.48% and 12.63%). The increased forest is mainly distributed in Jingxi Gou Township and Gonghe County. Unused land decreased by 267.31 km^2^ but its SLUDD was only −0.37% because the unused land area in the initial stage was large.

#### 3.1.2. Land Use and Land Cover Change for 2010–2020

Simulation results indicated that the comprehensive land use dynamic degree (CLUDD) in the Qinghai Lake region for 2010–2020 was 0.22%. The area of cropland decreased by 124.89 km^2^ (Table S3), and its SLUDD was −1.69%. The forestland increased by 62.24 km^2^ (SLUDD: 0.53%). The area of wetland decreased by 265.20 km^2^, and its SLUDD was 0.88%. Most of them were converted to unused land and grassland, distributed in Suli Township of Tianjun County and Quanji Township of Gangcha County (Figure 3c,d). The area of grassland increased by 456.41 km^2^. The increased grassland was mainly distributed in the north area of the Qinghai Lake (Figure 3c,d). The area of unused land decreased by 146.73 km^2^ and construction land increased by 15.02 km^2^. The area of waterbody remained stable in general.

### 3.2. Ecosystem Service Values under the Influence of Land Use and Land Cover Change

#### 3.2.1. Ecosystem Service Values for 1990–2020

**Figure 4:**
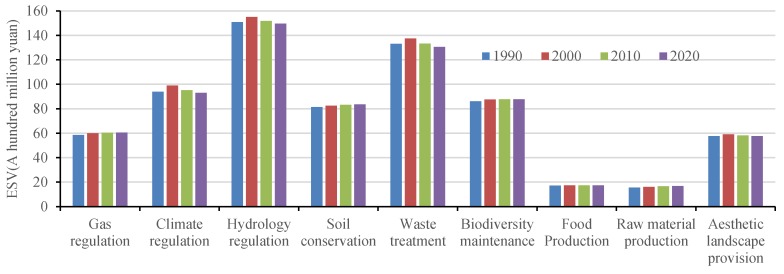
Ecosystem service values (ESV) for 1990–2020 in the Qinghai Lake region. Data were calculated based on Equations (4)–(6).

Using the average grain yield of grain crops and the average grain price per hectare of Qinghai Province [41,42,43], ESV for different land use types and for each function were calculated for 1990, 2000, 2010, and 2020 based on Equations (4)–(6) (Table 2 and Figure 4).

The total ESV in the Qinghai Lake region shows an increasing trend with fluctuations. It increased from 694.50 billion Yuan in 1990 to 714.28 billion Yuan in 2000 and then decreased to 703.59 billion Yuan. Simulation results indicate that the total ESV in 2020 will continue decreasing to 696.72 billion Yuan with a decrease of 1.01% compared to 2010. In terms of land use types, grassland, waterbody, and wetland provide most of the ESV of the study area, which service value accounts for more than 95% of the total value. From 1990 to 2010, the trend of ESV provided by cropland and grassland showed a similar pattern with the total ESV. The ESV provided by woodland and unused land has been increasing over 1990–2010, while the ESVs of wetlands and waterbody decreased with fluctuations. The results for 2020 indicated that the ESV of wetland would decrease and ESVs of forest, grassland, and waterbody would increase.

Among all ecosystem services (Figure 4), the values of hydrology regulation and waste treatment are the top two functions that account for more than 40% of the total value. Climate regulation, biodiversity conservation, and soil conservation accounted for about 35% of the total value. The value of other ecosystem services is low. For 1990–2000, the values of all types of ecosystem services increased, especially climate regulation, hydrological regulation, and waste treatment. However, from 2000 to 2010, the ESVs declined. The simulation shows that, by 2020, the values of gas regulation (means regulation of atmospheric chemical composition), soil conservation, and raw material production would increase significantly, and the value of other services would remain stable or decline slightly.

#### 3.2.2. Spatial Pattern of Ecosystem Service Values

**Figure 5:**
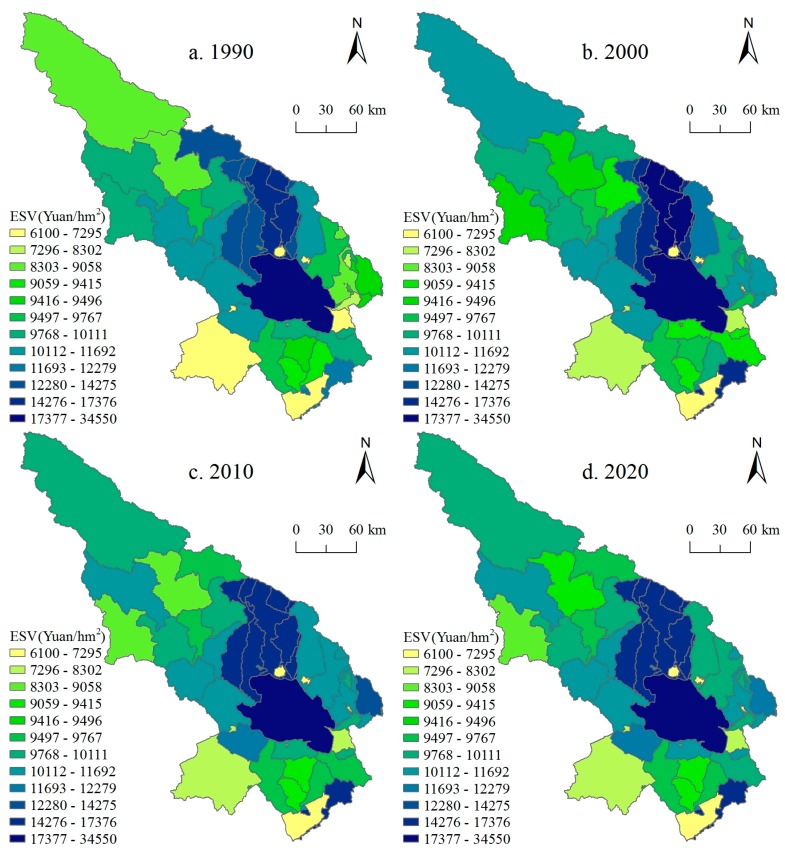
Town scale ecosystem service value (ESV) in the Qinghai Lake region for 1990–2020. Data were calculated based on Equations (4)–(6).

Figure 5 shows the ESV of per unit area at the town scale in Qinghai Lake regions for 1990–2020. There is spatial heterogeneity in ESV under the influence of land use change, but the change is not obvious for 1990–2020. Overall, the high value area of ESV was mainly located in central and north of the study regions as well as the Qinghai Lake region. The southwest and northwest regions of the study area have low ESV.

In terms of changes in the ESV of per unit area at a town scale, the regions with high variation for 1990–2020 include the northwest regions, the Qinghai Lake and its surrounding regions (Figure 5). For 1990–2010, the ESV of per unit area for the towns in the eastern Qinghai Lake regions increased substantially. The rapid development of animal husbandry in these areas drives the conversion of unused land to grassland. In addition, the Longyangxia, Quanji, Jiermeng, and Suli towns also experienced rapid increase of the ESV. However, the ESV of per unit area for Muli and Shengge towns decreased. For 2010–2020, the variations of ESVs per unit area follow similar spatial patterns of that for 1990–2010 (Figure 5c,d).

**Figure 6:**
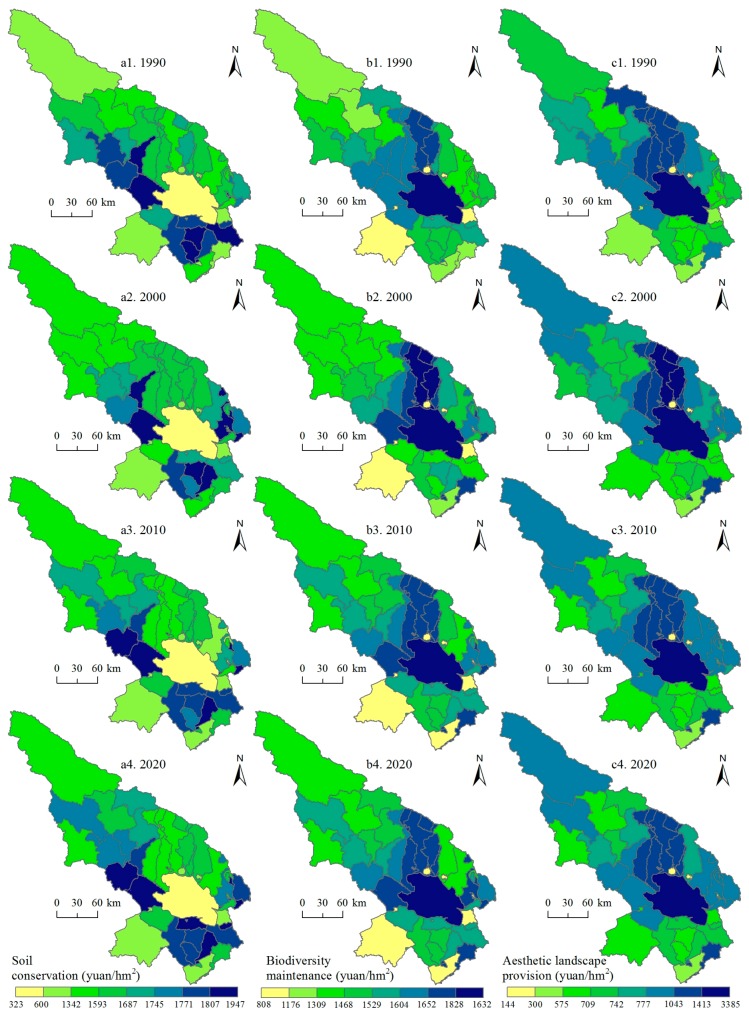
Soil conservation service, biodiversity maintenance service, and aesthetic landscape provision service in the Qinghai Lake region for 1990–2020. Data were calculated based on Equations (4)–(6).

**Figure 7:**
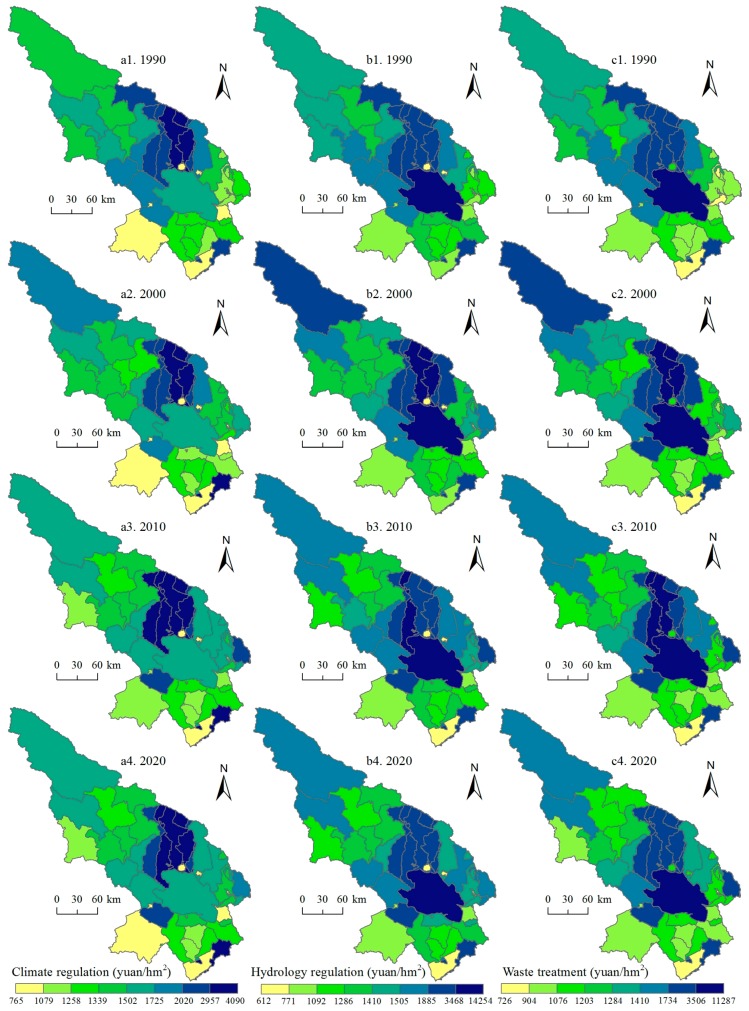
Climate regulation, hydrology regulation, and waste treatment services in the Qinghai Lake region for 1990–2020. Data were calculated based on Equations (4)–(6).

Subsequently, we paid attention to two support services (soil conservation and biodiversity maintenance), one culture service—aesthetic landscape provision (Figure 6), and three regulation services (climate regulation, hydrology regulation, and waste treatment, Figure 7). Soil conservation service is high in the south and northwest of the Qinghai Lake regions and the east regions saw increases of soil conservation service for 1990–2020. The hotspots of biodiversity maintenance include the Qinghai Lake and its north regions. We can also see that the northwest experienced decreased of biodiversity maintenance service. As a famous tourist area, the Qinghai Lake and its north regions own high aesthetic landscape provision service, and the value of this service increased slightly for 1990–2020. The hotspots of the hydrology and waste treatment services are the Qinghai Lake and its north regions, but, for the climate regulation service, the north of the Qinghai Lake saw high values.

### 3.3. Sensitivity of Ecosystem Service Values to Land Use and Land Cover Change

The SI of the three periods for the entire study region were 0.53, 0.32 and 0.80, respectively. From the first decade to the second, the SI decreased slightly, which means the ecological environment recovered slightly. However, from the second decade to the third one, the SI increased by 151.67%, indicating that the ecological environment would degrade during 2010–2020.

**Figure 8:**
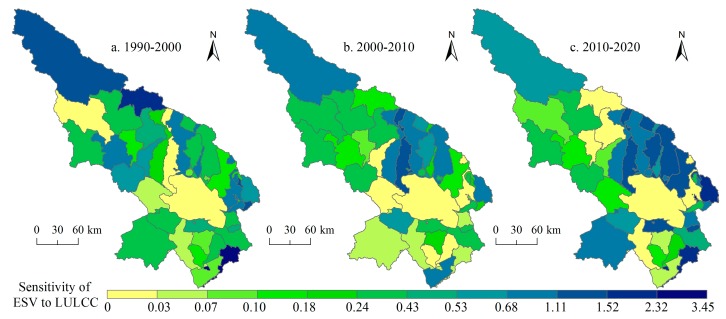
Sensitivity index of ecosystem service values (ESV) to land use and land cover change (LULCC) in the Qinghai Lake region. Data were calculated based on Equation (7).

The spatial patterns of the SI for 1990–2000, 2000–2010, and 2010–2020 in the Qinghai Lake region are depicted in Figure 8. The towns with high SI are mainly located in the Suli Town, Muli Town, and Shengge Town of Tianjun County, the Shaliuhe Town and Ike-Wulan Town of Gangcha County, the Xihai Town, Halejing Mongolian Town, Sanjiaocheng Town of Haiyan County. From the perspective of changes in the spatial pattern of SI, the SI of the northwest and south of the study area decreased from the first period to the second while the SI of the north of the Qinghai Lake increased. From the second period to the third one, the SI increased obviously for the entire study area, especially for the north and east of the Qinghai Lake.

**Figure 9:**
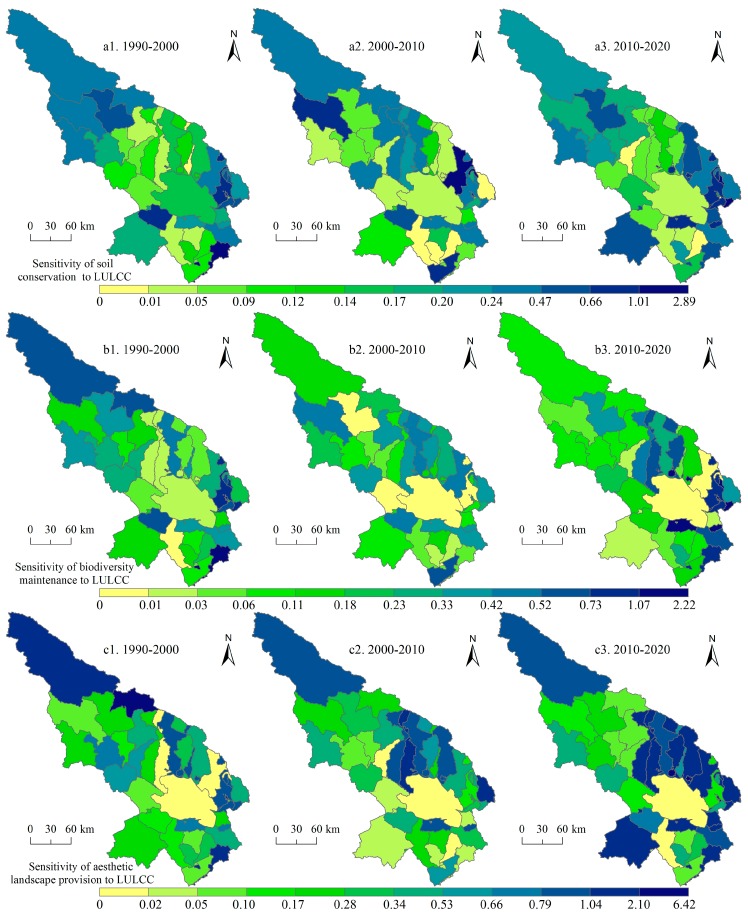
Sensitivity index of soil conservation service, biodiversity maintenance service, and aesthetic landscape provision service to land use and land cover change (LULCC) in the Qinghai Lake region for 1990–2000, 2000–2010, and 2010–2020. Data were calculated based on Equation (7).

**Figure 10:**
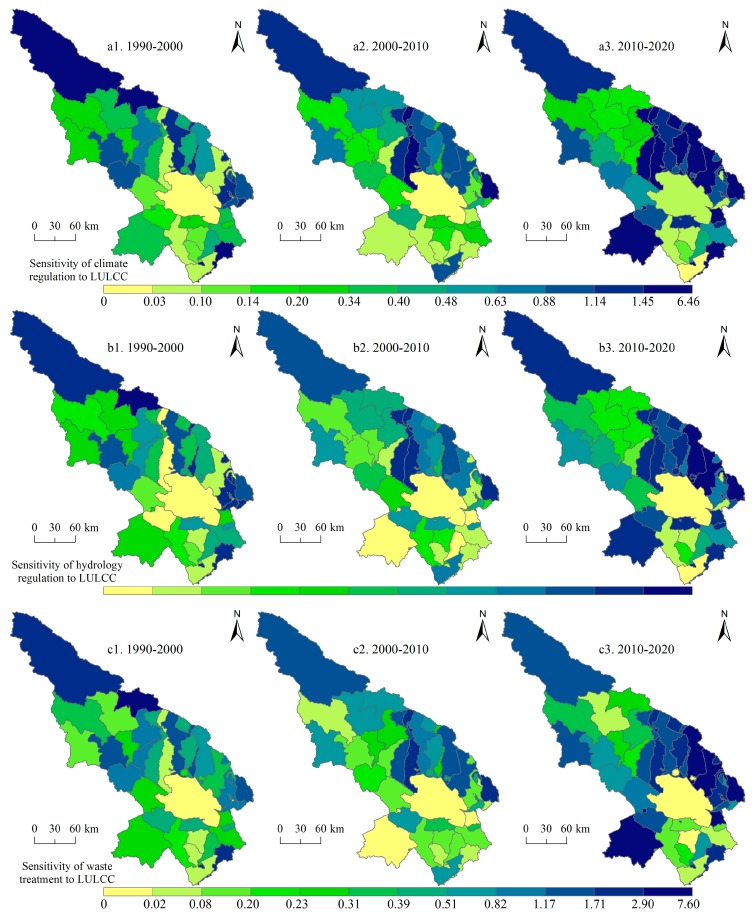
Sensitivity index of climate regulation, hydrology regulation, and waste treatment services to land use and land cover change (LULCC) in the Qinghai Lake region for 1990–2000, 2000–2010, and 2010–2020. Data were calculated based on Equation (7).

In addition, the SIs of two support services (soil conservation and biodiversity maintenance), and one culture service—aesthetic landscape provision were illustrated for the three study period (Figure 9). For the two support services, the SI decreased in the Qinghai Lake and its surrounding regions on the whole for 1990–2000 and 2000–2010, which is a good thing, but for 2010–2020, some towns experienced increase of SI. In terms of the aesthetic landscape provision, we can see that the SI increased obviously in the surrounding regions of the Qinghai Lake for the whole study period, which will potentially reduce the attractions of these regions to tourists. Subsequently, the SIs of the three regulation services (climate regulation, hydrology regulation, and waste treatment, Figure 10) were analyzed. It can be seen that the SI of the three regulation services increased substantially in the north of the Qinghai Lake and the Qieji town from 2000–2010 to 2010–2020. Some measures must be taken to lower the SIs of these places for human well-beings.

Furthermore, the number and area of town units and their proportion to the entire study area were calculated in terms of changes of SI over the three periods (Table 3). The number of the towns for which SI decreases from 1990–2000 to 2000–2010 (23 towns) is more than the number of the towns that experienced an increase in SI (only 14). The corresponding area proportions of these towns are 70.08% and 29.92% respectively. It indicates that the environmental conditions of most towns improved during 2000–2010. From 2000–2010 to 2010–2020, the situation is reversed. There are 29 towns with increased SI and only nine towns with decreased SI. The area proportion for the former is 60.19% and for the latter is 39.81%. These results indicate that the ecosystems of most regions in our study area are becoming more sensitive to human land use activities from 2010 to 2020.

## 4. Discussion

### 4.1. Analysis on Driving Forces of Land Use and Land Cover Change

**Figure 11:**
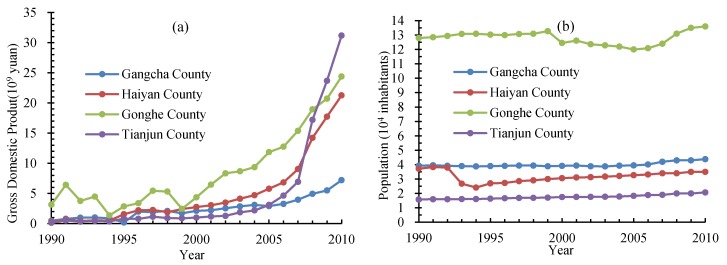
(**a**) Gross domestic product and (**b**) population of the four counties surrounding the Qinghai lake region for 1990–2010. Data were cited from Qinghai statistics yearbook for 1990–2010.

The main land use type of the Qinghai lake region is grassland. Our results indicated that the area of grassland decreased for 1990–2000 and after that it increased. Land use change is driven by anthropogenic and natural factors. For 1949–1987, the population of this region increased gradually, and the number of livestock increased by three times [49]. For 1990–2000, the gross domestic product (GDP) and population of the four counties increased slowly in general (Figure 11). In the end of year 2000, the population of the four counties of the Qinghai lake region is 211.8 thousand (Figure 11) and the number of livestock is 3.03 million [42], which led to the degradation of grassland ecosystem because of overgrazing. In addition, owing to the rise of rapeseed oil price since the mid-1990s, a large area of grassland was reclaimed to plant rape flower, which contributed to the desertification of grassland.

Since 2000, the central and local government implemented some ecological engineering on the Tibetan Plateau, including Grain for Green (forest and grassland), to restore the environment of Qinghai Lake and its surrounding regions [8]. A series of ecological engineering programs, which was called as a 10-year project, were implemented further after 2008. The project includes a suite of tasks, for example, the sweeping program, which encourages to reduce grazing on grasslands, control rodents and insect pests that damage alpine meadows, protect wetlands, prevent desertification, plant trees and shrubs, and protect biodiversity. Almost 1.6 billion Yuan was poured into the program by the government. The increase of grassland area is consistent with the implementation period of ecological engineering on the Tibetan Plateau. Thus, the ecological engineering can be seen as one of the main driving forces for increase in grassland area [50]. Although the GDP, population, and the number of livestock increased too for 2000–2010, the ecological conditions of here restored overall.

Additionally, climate warming also contributes to land use and land cover change in this region. According to the data of meteorological stations in Tianjun and Gangcha counties, the annual average temperature of the two stations for 1990, 2000, and 2010 are −0.14, −0.47 and 1.04 °C respectively, and the annual average precipitation are 367.60, 336.30 and 416.30 mm. The warming-drying tendency for 1990–2000 and the warming-wetting tendency for 2000–2010 contributed to the decrease of grassland area for the first period and the increase for the second period.

### 4.2. Identification of Ecologically Sensitive Areas to Land Use and Land Cover Change

According to the development plan, Four zones, Two belts, and One line Development Plan in Planning of Main Functional Area in The Qinghai Province (2008–2020), the Qinghai Lake region was identified as a modern high-efficiency animal husbandry production base and a demonstration area for human-nature harmony. Therefore, more attention should be paid to ecologically sensitive areas to LULCC.

Sensitivity analysis of ESV to LULCC indicates that the sensitive areas include three towns of the Tianjun County, two towns of the Gangcha County, and three towns of the Haiyan County. Reduction in land use intensity for protection and sustainability should be encouraged in these areas. For 2010–2020, the SI of the total study area would increase by 151.67%, is mainly distributed in the north and east of the Qinghai Lake. As “a jewel of the crown” on the Tibetan Plateau, the Qinghai Lake region is extremely sensitive, not only to climate changes but also to land use activities. As the ecologically most sensitive areas of the Qinghai Lake region, it must be protected with cautions in the process of economic development. In addition, under the influence of ecological restoration engineering, these regions still tend to be sensitive to human activities, implying that much work remains to be done to better understand its environmental characteristics for protection and land use planning.

### 4.3. Comparison with Previous Studies

In this study, ESV decreased from 2000 to 2010, which is consistent with the findings by Li et al. [51] that suggested the ESV in the Qinghai Lake region decreased from 2000 to 2008. Cao et al. [49] found that hydrology regulation and waste treatment are the two top ecosystem services, which is consistent with the outcomes of this study. In addition, compared with the previous studies concerning land use change [8] and its impact on the ESV of the Tibetan Plateau [10], this study both captures the land use/cover change and its effects on ESV for 1990–2010. The Markov-CA model is also used to simulate land use change and predict its impact on ESV for 2020, which is more useful for future land use planning and the development of ecosystem-related sustainable strategies.

Compared with the counties of the mid-east areas of China, the land areas of most counties in the Tibetan Plateau are large. Therefore, the assessment at a county scale is inadequate. In this study, by collecting higher resolution data, we assessed the LULCC-induced ESV and its sensitivities at township scale and specific analysis was done for two support services (soil conservation and biodiversity maintenance), one culture service (aesthetic landscape provision), and three regulation services (climate regulation, hydrology regulation, and waste treatment services). This will provide more scientific support for local or regional decision-making.

### 4.4. Uncertainty Analysis

First, in the process of simulating the land use/cover pattern for 2020, the Markov model is used as the land suitability map that only takes into account the historical trend of land use change. However, the effect of land change driving factors on land suitability is not taken into account. This will bring uncertainties to simulation outcome, which should be kept in mind by scholars for future studies and decision makers for land use planning. Specifically, the change tendency of land use area for 2010–2020 is extrapolated based on the area change tendency for 2000–2010, and the simulated land use/cover map for 2020 is more similar to the initial input map of 2010 than maps of 1990 and 2000, which means the simulation outcome could only capture the overall pattern, instead of the fine details. In the future, we will take anthropogenic and natural driving factors into consideration to derive the land suitability map and employ other simulation methods for further improvement [52].

The coefficients of Xie et al. [37] are applicable to the national scale. Although they were directly used in many regional scales [10,53], calibration before application will obtain more reliable results [49]. Because of the limited time and the cost constraints, this study did not localize the coefficients in the Qinghai Lake. It may overestimate the ESVs of the Qinghai Lake region since the biomass of here is lower than the average biomass of China [37], especially for waste regulation services, which should be considered when using the ESV results to inform decision-makers.

In addition, this study only assessed the impact of LULCC on ESV. The climate of the Tibetan Plateau has undergone significant changes over the decades [54], which is bound to exert an impact on ESV. Using spatially explicit models, for example, the Integrated Valuation of Ecosystem Services and Tradeoffs (InVEST) model [4,22,55], to assess the joint impact of LULCC and climate change on ESV will be one of our focus in future studies [56].

## 5. Conclusions

By interpreting remote sensing images and utilizing Markov-CA simulation, we analyzed the LULCC characteristics in the Qinghai Lake region. We then assessed the response of ecosystem services to LULCC at a township scale. For 1990–2010, the area of construction land, forest, grassland, and cropland increased while the area of unused land and wetland decreased. Markov-CA simulation results suggested that the area of grassland, forest, and construction land will increase and the area of cropland and unused land will decrease for 2010–2020. Under the influence of LULCC, the ESV in the Qinghai Lake region increased first and then decreased from 1990 to 2010. For 2010–2020, the ESV would decrease slightly. The values of hydrology regulation and waste treatment are the top two functions that account for more than 40% of the total value. In terms of the spatial patterns of the ESV, the towns in the northwest of the study region and the Qinghai Lake have high ESVs while the towns in the west and south of the study area have low ESVs. The regions with high variation of ESV are located in the north of the Qinghai Lake. The towns with high SI are located in the middle and northwest of the study area and the towns in the north of the Qinghai Lake experience increase in SI from 2000–2010 to 2010–2020.

It should be mentioned that this study did not explicitly take the land change driving forces into account in simulating LULCC and also did not localize the national scale ESV estimation coefficients for the study area. Future work will consider overcoming these limitations.

## Figures and Tables

**Table 1:** Land use transition matrix for 1990–2000 in the Qinghai Lake region (area unit: km^2^).

Land Use Type	Cropland	Forestland	Grassland	Wetland	Waterbody	Construction Land	Unused Land	Total
Cropland	514.51	0.14	130.72	9.82	1.06	11.97	17.30	685.52
Forestland	0.15	492.14	266.35	19.88	0.00	0.02	3.70	782.24
Grassland	384.13	507.59	34,705.90	1173.80	13.23	20.34	2319.89	39,124.88
Wetland	0.22	9.24	1009.59	1959.90	1.74	1.17	71.13	3052.98
Waterbody	0.01	0.14	47.23	25.94	4287.87	0.05	22.64	4383.89
Construction land	2.63	0.00	1.14	0.08	0.00	3.60	0.00	7.45
Unused land	11.35	15.43	2585.72	303.90	11.35	0.68	4871.10	7799.54
Total	913.00	1024.68	38,746.64	3493.31	4315.26	37.85	7305.76	55,836.49

**Table 2:** Ecosystem service value of different land use types in the Qinghai Lake region for 1990–2020 (unit: 10^9^ Yuan).

Year	Cropland	Forestland	Grassland	Wetland	Waterbody	Construction Land	Total
1990	4.37	17.75	368.35	134.90	160.39	8.75	694.50
2000	5.82	23.25	364.79	154.35	157.88	8.19	714.28
2010	4.72	26.68	371.05	134.29	158.96	7.89	703.59
2020	3.93	28.09	375.41	122.50	159.07	7.72	696.72

**Table 3:** Changes of sensitivity index for 1990–2020 in terms of number and area of townships. Data were calculated based on Equation (7).

Compare Periods	Changes of Sensitivity Index	Number of Townships		Area of Townships	
		Number	Proportion (%)	Area (10^4^ km^2^)	Proportion (%)
1990–2000 and 2000–2010	Decrease	23	62.16%	39.07	70.08%
	Increase	14	37.84%	16.68	29.92%
2000–2010 and 2010–2020	Decrease	9	24.32%	22.20	39.80%
	Increase	28	75.68%	33.56	60.20%

## Supplementary Materials

The following are available online at www.mdpi.com/1660-4601/14/7/818/s1. Table S1: Ecosystem service value equivalent factors of per unit area in China. Table S2: Land use transition matrix for 2000–2010 in the Qinghai Lake region (area unit: km^2^). The row is 2000 and the column is 2010. Table S3: Land use transition matrix for 2010–2020 in the Qinghai Lake region (area unit: km^2^). The row is 2010 and the column is 2020.
