# Supplementary material for: Land Use and Land Cover Change in the Qinghai Lake Region of the Tibetan Plateau and Its Impact on Ecosystem Services
Source: Int J Environ Res Public Health. 2017 Jul 21;14(7):818. doi: 10.3390/ijerph14070818 (PMC5551256; doi:10.3390/ijerph14070818)
Supplement: Supplementary file 1 [file ijerph-14-00818-s001.pdf]

International Journal of
*Environmental Research
and Public Health*

MDPI

Supplement Materials

Land Use and Land Cover Change in the Qinghai Lake Region of the Tibetan Plateau and Its Impact on Ecosystem Services

Jian Gong [1,2,*], Jingye Li [1], Jianxin Yang [1], Shicheng Li [1] and Wenwu Tang [3,4]

Table S1. Ecosystem service value equivalent factors of per unit area in China (Xie et al., 2008).

Primary Type	Secondary Type	Forestland	Grassland	Cropland	Wetland	River/Lake	Desert
Provision	Food Production	0.33	0.43	1.00	0.36	0.53	0.02
	Raw material production	2.98	0.36	0.39	0.24	0.35	0.04
Regulation	Gas regulation	4.32	1.50	0.72	2.41	0.51	0.06
	Climate regulation	4.07	1.56	0.97	13.55	2.06	0.13
	Hydrology regulation	4.09	1.52	0.77	13.44	18.77	0.07
	Waste regulation	1.72	1.32	1.39	14.40	14.85	0.26
Support	Soil conservation	4.02	2.24	1.47	1.99	0.41	0.17
	Biodiversity maintenance	4.51	1.87	1.02	3.69	3.43	0.40
Culture	Aesthetic landscape provision	2.08	0.87	0.17	4.69	4.44	0.24

Table S2. Land use transition matrix for 2000–2010 in the Qinghai Lake region (area unit: km^2). The row is 2000 and the column is 2010.

Land use type	Cropland	Forestland	Grassland	Wetland	Waterbody	Construction Land	Unused Land	Total
Cropland	609.35	1.46	289.49	0.55	0.10	2.09	9.97	913.00
Forestland	0.40	444.74	535.45	24.14	0.00	0.63	19.33	1024.68
Grassland	109.56	709.31	34587.06	935.78	26.98	43.67	2334.29	38746.64
Wetland	4.27	7.80	1239.54	2012.70	17.29	3.12	208.59	3493.31
Waterbody	0.00	0.00	17.41	2.13	4284.17	0.00	11.54	4315.26
Construction Land	0.89	0.00	0.87	0.00	0.00	36.09	0.00	37.85
Unused Land	16.60	12.72	2741.49	64.00	16.15	0.06	4454.74	7305.76
Total	741.06	1176.02	39411.30	3039.29	4344.69	85.66	7038.45	55836.49

Table S3. Land use transition matrix for 2010–2020 in the Qinghai Lake region (area unit: km^2). The row is 2010 and the column is 2020.

Land use type	Cropland	Forestland	Grassland	Wetland	Waterbody	Construction Land	Unused Land	Total
Cropland	613.28	0.11	125.20	0.36	0.00	0.80	1.17	740.92
Forestland	0.04	1059.72	84.49	22.09	0.00	0.16	9.32	1175.81
Grassland	0.53	170.89	39099.66	89.54	1.43	19.68	36.32	39418.06
Wetland	0.40	4.72	261.69	2658.95	3.69	0.21	107.97	3037.64
Waterbody	0.00	0.00	1.60	0.14	4341.48	0.00	1.47	4344.69
Construction Land	1.34	0.10	4.10	0.27	0.01	79.49	0.33	85.64
Unused Land	0.44	2.51	297.74	1.08	1.23	0.31	6730.41	7033.73
Total	616.03	1238.05	39874.47	2772.44	4347.85	100.66	6887.00	55836.49

References

1. Xie, G.D.; Zhen, L.; Lu, C.X.; Yu, X.; Cao, C. Expert knowledge based valuation method of ecosystem services in China. *J. Nat. Resour.* **2008**, *23*, 911–919.
